# Pulmonary hernia: Case report and review of the literature

**DOI:** 10.1002/rcr2.354

**Published:** 2018-10-02

**Authors:** Chiara Scelfo, Chiara Longo, Marina Aiello, Giuseppina Bertorelli, Ernesto Crisafulli, Alfredo Chetta

**Affiliations:** ^1^ Department of Medical Specialties, Pneumology Unit Arcispedale Santa Maria Nuova‐IRCCS, Azienda USL di Reggio Emilia Reggio Emilia Italy; ^2^ Department of Medicine and Surgery, Respiratory Disease and Lung Function Unit University of Parma Parma Italy

**Keywords:** Pulmonary hernia, thoracic surgical intervention

## Abstract

Pulmonary hernia (PH) is an uncommon condition. We report a case of PH secondary to thoracic surgical intervention. In addition to the rarity, the peculiarity of the case is given by the clinical course as it is characterized by a clinical latency before the onset. The patient showed risk factors such as obesity and poliomyelitis infection sequelae. We also reviewed the literature about this topic.

## Introduction

Hernia is a general term used to describe a bulge or protrusion of an organ through the structure or muscle that usually contains it. There are many different types of hernias.

Pulmonary hernia (PH or pneumocele) is an uncommon condition. It refers to part of a lung pushing through a tear, or bulging through a weak spot, in the chest wall. In the majority of reported patients, lung hernias are the result of injury or trauma to the chest area, such as a fall or car accident, but there are a number of other possible causes.

This case report describes a case of lung hernia after thoracic surgery, along with review of the literature.

## Case Report

**Figure 1:**
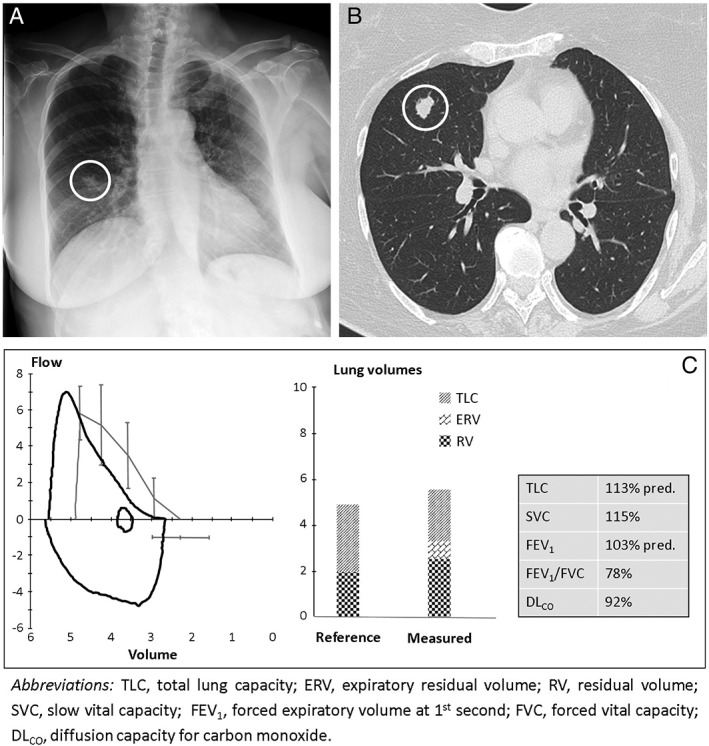
Chest X‐ray and computed tomography (CT) showing pulmonary nodule (A and B, white circles). Lung function testing at baseline (C).

A 65‐year‐old woman, obese (body mass index‐BMI 33.2 kg/m^2^), with no history of smoking habit underwent a nodulectomy for the incidental finding of a pulmonary nodule on chest radiography and on computed tomographic (CT) scan (Fig. 1A and B, white circles), with subsequent histological diagnosis of atypical mycobacterial infection.

She had a clinical history of poliomyelitis at 9 years old, resulting in right hemiplegia and a hysterectomy including the ovaries at 45 years old. Before surgery, her clinical condition was normal; the functional respiratory tests showed an increase in residual volume, probably caused by an expiratory muscle weakness due to the poliomyelitis in the past (Fig. 1C).

**Figure 2:**
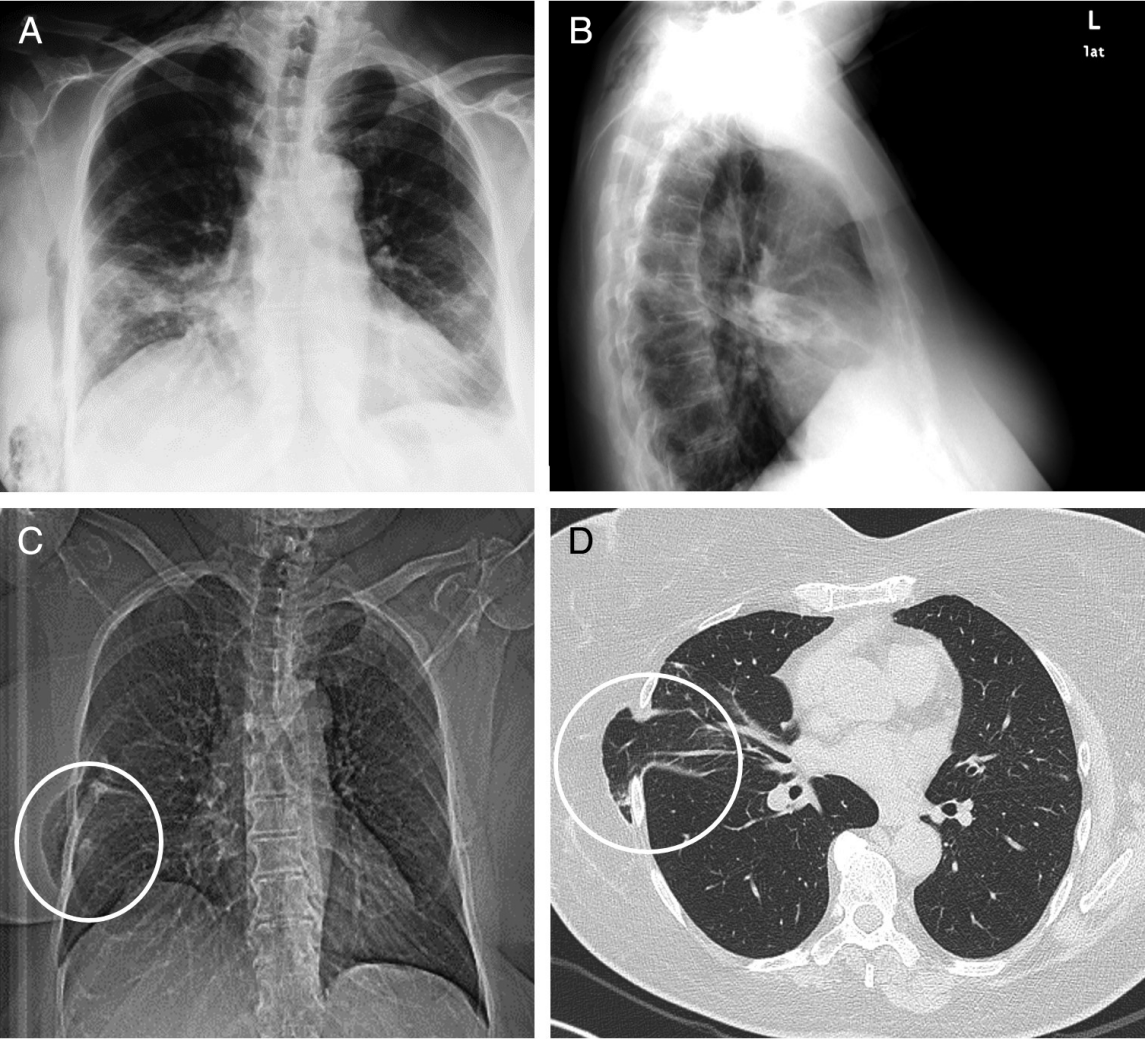
Chest X‐ray after nodulectomy surgery: subcutaneous, right, parietal emphysema (A and B). Chest computed tomographic (CT) scan showing lung hernia (C and D, white circles).

**Figure 3:**
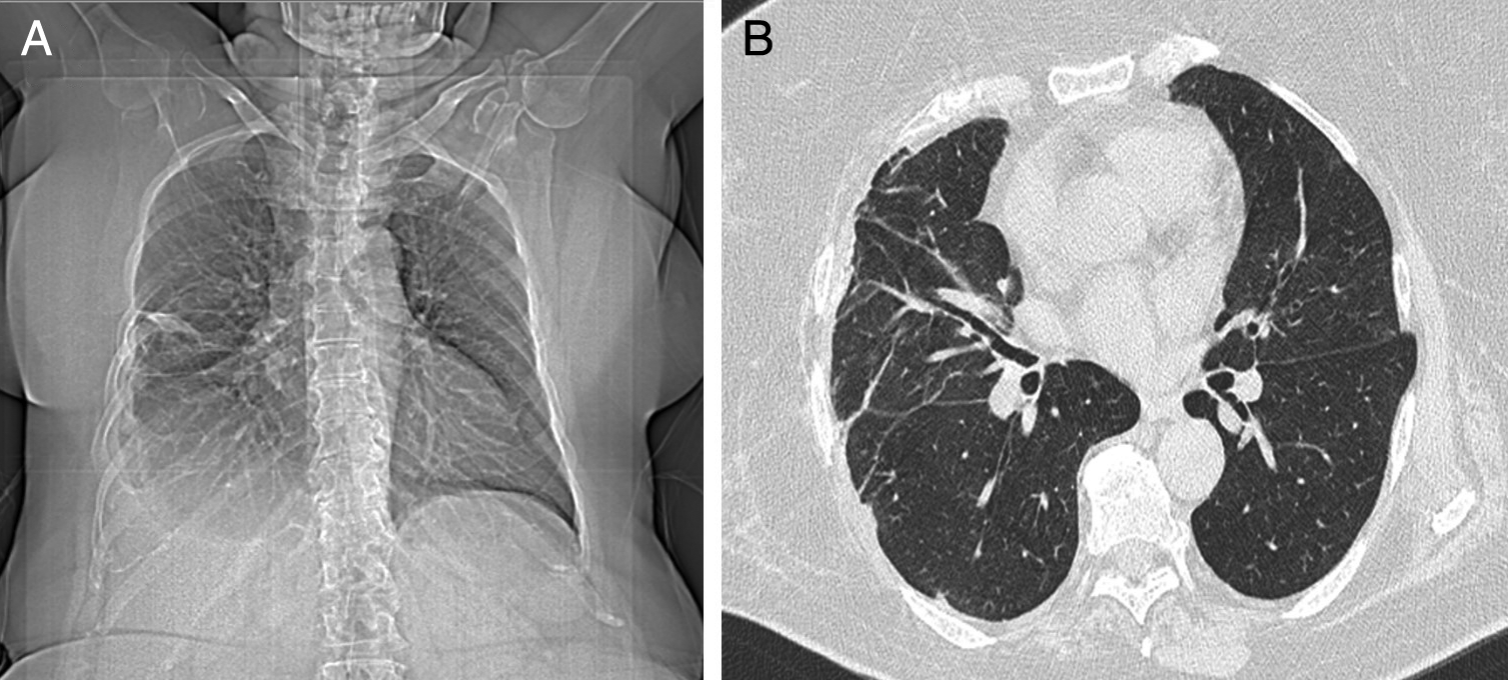
Chest computed tomography (CT) showing the radiographic resolution after surgical reduction (A and B).

The postoperative course was complicated by subcutaneous, right, parietal emphysema, which extended up to the neck (Fig. 2A and B). Five months later, the patient underwent a chest CT scan for the subsequent appearance of chest pain, dyspnoea, and asthenia; the lung hernia (67 X 13 mm) was revealed as a hole of 35 mm in the space between the fifth and the sixth rib of the right chest wall (Fig. 2C and D, white circles). Next, the lung hernia was reduced surgically, resulting in a right pleural effusion and significant subcutaneous emphysema. The almost complete resolution of the clinical and radiographic (Fig. 3A and B) conditions occurred after a year and a half, during which time the patient underwent periodic follow‐up.

## Discussion

The occurrence of hernial protrusions of the lung is exceedingly rare, and the literature on this topic is scarce. Interestingly, PH was first described by Dr. Roland in 1499 1. Next, the most widely accepted classification is that of Morel‐Lavallee 2 based on both the aetiology (congenital or acquired) and anatomic location (cervical, thoracic, or diaphragmatic).

Congenital hernias result from the attenuation of the endothoracic fascia. They occur either at the thoracic inlet or at the intercostal spaces, where weakness of the fascia is usually combined with absence of the intercostal muscles 3. However, approximately 80% of reported cases of lung hernia are acquired 4. They were further divided into traumatic, pathologic, and spontaneous. The majority of these cases result from trauma to the chest (penetrating or blunt) or from preceding operative procedure with inadequate closure of the chest wall 5, 6. Traumatic hernias may appear immediately after injury or be delayed for years 7.

Patients at the highest risk of a lung hernia seem to be those with elevated intra‐thoracic pressure, such as patients with morbid obesity or end‐stage chronic obstructive pulmonary disease 8. Other risk factors include tissue weakness or poor healing from malnutrition, steroids, diabetes, or other comorbidities.

The location of a PH is usually anterior, especially in the lower intercostal spaces; the next most common position seems to be lateral, with the rarest position being posterior. This is due to anatomic conditions; in fact, the anterior inferior part of the chest has much less muscular support than the anterior superior part, the sides, or the posterior part, and the lower costal cartilages are more widely separated than the rest 9.

In our case, lung herniation after thoracic surgery was likely due to the anatomical weakness of the intercostal muscles following surgery. With respect to the anatomy of the intercostal spaces, there are three muscles that cover its extension: the external, the medial, and the internal intercostal muscle (disposed from the surface to the deep layer). Their incomplete distribution within the spaces leads to areas of potential weakness 10. The anterior edge of the space (adjacent to sternum bone) and the posterior edge (adjacent to the vertebral spine) are areas of vulnerability because they are covered by only one of the three muscles mentioned 7. This anatomic condition could worsen in the presence of the above‐mentioned clinical conditions, such as obesity and poliomyelitis infection sequelae. The obesity causes an elevated intra‐thoracic pressure, while the poliomyelitis infection explains the weakness of the thoracic wall. It is of note that, although the majority of the PHs are produced in the parasternal region (anterior extreme of intercostals space) 11, 12, in our patient, the hernia was in the posterolateral region.

PH can be asymptomatic. However, the classic clinical history is acute chest wall pain after coughing or sneezing. Acute evaluation shows rib fractures on chest film and chest wall ecchymosis on physical examination. The diagnosis should be confirmed by functional respiratory exam and thorax imaging. A computed tomographic scan is necessary to assess the exact location and size of the defect 13. Treatment may be conservative or surgical, but as experience is limited, no long‐term results have been reported. Asymptomatic hernias require no treatment, while increasing size, pain, and the difficulty to reduce the hernia, are the main indications for operation. Our patient opted for surgery because of persistent chest pain and dyspnoea, without relapse. To repair the anatomical defect, Munnell 14 recommended the use of autologous tissues whenever possible. However, synthetic materials (e.g. Dacron, Teflon) were acceptable when local tissues were not available.

In conclusion, the clinical case that came to our attention was secondary to thoracic surgical intervention. In addition to the rarity, the peculiarity of the case is given by the clinical course; it was characterized by a clinical latency before the onset and complete resolution in an obese patient with a history of poliomyelitis. However, PH should be considered a post‐thoracic surgery complication; the surgery treatment should be considered in symptomatic patients and in those with severe complications.

## Disclosure Statement

Appropriate written informed consent was obtained for publication of this case report and accompanying images.
